# Industrial air pollution in rural Kenya: community awareness, risk perception and associations between risk variables

**DOI:** 10.1186/1471-2458-14-377

**Published:** 2014-04-17

**Authors:** Eunice Omanga, Lisa Ulmer, Zekarias Berhane, Michael Gatari

**Affiliations:** 1 Impact Research and Development Organization, Kisumu, Kenya; 2 School of Public Health, Drexel University, Philadelphia, USA; 3 Institute of Nuclear Sciences and Technology, University of Nairobi, Nairobi, Kenya

**Keywords:** Environmental, Perception, Industrial, Air, Pollution, Risk, Rural

## Abstract

**Background:**

Developing countries have limited air quality management systems due to inadequate legislation and lack of political will, among other challenges. Maintaining a balance between economic development and sustainable environment is a challenge, hence investments in pollution prevention technologies get sidelined in favor of short-term benefits from increased production and job creation. This lack of air quality management capability translates into lack of air pollution data, hence the false belief that there is no problem. The objectives of the study were to: assess the population’s environmental awareness, explore their perception of pollution threat to their health; examine the association between specific health hazards.

**Methods:**

A cross-sectional study was implemented by gathering quantitative information on demographic, health status, environmental perception and environmental knowledge of residents to understand their view of pollution in their neighborhood. Focus group discussions (FGDs) allowed for corroboration of the quantitative data.

**Results:**

Over 80% of respondents perceived industrial pollution as posing a considerable risk to them despite the fact that the economy of the area largely depended on the factory. Respondents also argued that they had not been actively involved in identifying solutions to the environmental challenges. The study revealed a significant association between industrial pollution as a risk and, perception of risk from other familiar health hazards. The most important factors influencing the respondents’ pollution risk perception were environmental awareness and family health status.

**Conclusion:**

This study avails information to policy makers and researchers concerning public awareness and attitudes towards environmental pollution pertinent to development and implementation of environmental policies for public health.

## Background

Most developing countries, especially in sub-Saharan Africa, do not have air quality management systems because of inadequate legislation, budgetary constraints and lack of political will, among other things [1]. In these regions, there is a major challenge in maintaining a balance between economic development and a sustainable environment; hence investments in pollution prevention technologies like emission controls are commonly outweighed by the short-term benefits that accrue from increased production and job creation. The lack of air quality management capabilities in these regions translate into lack of air pollution data, which in most cases gives the false belief that it is not a problem. This however, is not the case and only further conceals a major public health crisis in the developing world.

More and more people are concerned about environmental hazards and the resultant adverse health effects on humans and the environment at local, regional and global levels [2-5]. For example, since the publication of Rachel Carson’s [6] book *Silent Spring*, public fear and concern over cancer from chemicals such as pesticide residues in food are on the increase. Pollution (air and water) adversely impact the environment and the effects frequently spread well beyond geographical borders. Since the establishment of the United Nations Environment program (UNEP) in 1972 with its headquarters in Nairobi, Kenya, national and international focus has been on environmental health effects of pollution, especially water pollution. While progress has been made in reducing sanitation related diseases like diarrhoea in the developing world, little has been done to combat the negative health effects of increased industrialization and resultant pollution.

A 2007 World Health Organization (WHO) report revealed huge inequalities on environmental impact on health in addition to demonstrating how public health could be improved by reducing environmental threats such as pollution, occupational hazards as well as climate and ecosystem changes [7]. This report revealed that globally, up to 13 million deaths could be averted annually through better environmental management and in some countries over 30% of the disease burden could be prevented. The WHO estimate of the burden of disease due to air pollution puts premature deaths attributed to air pollution at over 2 million, with residents of developing countries bearing half of this burden [8].

Pollution from industries negatively impacts the health of employees and neighbouring communities and the potential for adverse health outcomes is heightened when the industries are located in rural areas where the bulk of the population is vulnerable because of limited information about their rights and limited capacity to defend themselves or influence policy decisions. Rural communities are often overlooked by businesses and sometimes even the government. The deficient environmental health awareness coupled with lack of sustainable environmental health programs is a major challenge in most developing countries [8]. While majority of studies have been on urban populations, air pollution in rural communities is on the increase [9].

**Figure 1:**
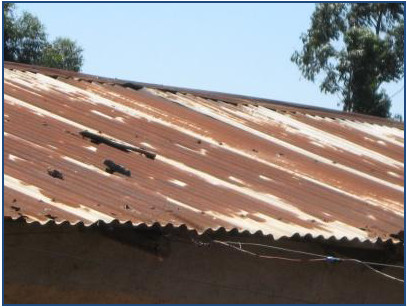
Corrosion on corrugated iron roof sheets close to factory due to air pollutions.

To date, estimates of air pollution health effects in the developing world rely mainly on data from research conducted in the developed world, specifically North America & Europe which is then extrapolated, yet the characteristics of the ambient pollutants and the environmental conditions, in most cases differ a great deal [10]. Besides, the prevailing health conditions of the population in the developing world differ from those in the developed world and influence therefore health outcomes are influenced differently. In Kenya, a few studies have shown how air pollution has, and continues to adversely affect human health and the built environment (Figure 1) and the ecosystem in general [1,11-14]. This is because for a long time there was no specific administrative or legislative framework within which to articulate and execute air quality management in Kenya. With the passage of the Environmental Conservation and Management Act of 1999 [15], and the creation of the National Environmental Management Authority (NEMA), Kenya’s potential to manage air quality has improved to a limited extent. However, the NEMA administrative framework and professional capacity continues to be expanded hence the urgent need for locally generated scientific data.

This study examined the perception of residents neighbouring a factory situated in a rural township in Kenya. It contributes to the literature on environmental risk perception by addressing environmental risks in a local cultural and social context. Respondents reflected on their perceived health threats and relations to the manufacturing industry in their neighbourhood. This research avails useful information to all the stake holders (community members, industry and government regulators) who may be involved in discussions over existing (or non-existing) environmental threats. It presents the situation as seen by the communities and therefore provides a framework for further research with regard to protection of the public health from industrial pollution in Kenya. Perception studies reveal unique elements such as cultural or local contexts of certain environmental issue, thereby expanding the range of sources for decision making in assessing risk factors and possible mitigation. The use of participatory approach combined with respondents’ interest in the study topic, helped to increase trust in this research work and confidence in the data collection process.

## Methods

### Study location and motivation

Four factors motivated the selection of the town for this study: (i) of the extensive media publicity on both air and water pollution including damage to the built environment (Figure 1); (ii) the presence of the factory and its being the largest employer in the area and therefore a backbone of the region’s economy; (iii) it represented a typical example of a rural based large manufacturing industry and therefore presented an opportunity to survey the neighboring residents’ perception of pollution to guide the development of applicable and appropriate policy options; (iv) no community level studies on pollution have been carried out before in the area. It is prudent to point out that even though the study was designed to gather residents’ views while the factory was operational, the factory closed down while ethics approval was being awaited (four months before the study commenced) due to liquidity problems.

### Characteristics of the industry

About 90% of toxic emissions from paper mills go into the atmosphere while 10% ends up in the water [16]. Sources of air pollutants from the industry include: power generation plant, boilers, bleaching plants and caustic soda/chlorine plant. Pollutants include particulate matter, chlorine, sulfur dioxides, hydrogen sulfides, carbon dioxide, carbon monoxides and nitrous oxides. Hydrogen sulfide (HS) in addition to being toxic has a very foul odor. Air pollutants of concern are particulate matter, hydrogen sulfide as well as sulfur and nitrate oxides. The vulnerability and health risks for communities neighboring industrial facilities are compounded by the possibility of an industrial accident, as was experienced in Bhopal, India in 1984 and Chernobyl, USSR in 1986 among others.

### Study design

This was a descriptive cross-sectional study in one of the seven Provinces of Kenya where residents living within a five-kilometer radius of the paper mill were interviewed on their pollution and health perceptions. The sample was stratified and proportionally allocated to include key informants of the stake holders in the community as well as the general population. Community gatherings (barazas) were used as the main forum for informing the community about the study. Such a system was successfully utilized by Mwanthi and Kimani [17] in their study of agrochemical handling and response in a rural community in Kenya. The purpose of the study and the community’s role in this research was first explained to the key leaders and then to the community at large. Prospective respondents were informed of the study and assured of confidentiality.

### Sample size determination and sampling procedure

The sampling frame was a random sampling of households within a 5-km radius of the industry and the formula below was used to determine the minimum sample size:

$$n=\frac{t^{2}\times \mathrm{pq}}{d^{2}}$$

where: *n* = the desired sample size (if the target population is greater than 10,000),

*t* = critical value for the desired confidence level (alpha),

*p* = proportion in the target population estimated to have characteristics being measured,

*q* =*1*-*p*, and *d* = desired precision.

Since no estimate was available for the expected proportion of the target population which had the characteristics of interest then, 50% (*p* = 0.5) was used and confidence level was set at with 95% for which *t* = 1.96 resulting in sample size of 384.

An additional 10% was added to take care of non-respondents that resulted with final sample size of 423.

The study area comprised of five sub-locations (clusters) which formed the strata. Proportionate stratified sampling was used to ensure the five clusters were adequately represented in the sample.

Rural Kenyans have structures through which official and community information is disseminated. The smallest unit is the village headed by a village elder. A number of villages form a clan and in some cases a group of ten villages form an administrative group known as *mji kumi* (ten homes). Village or clan leaders are usually elected by the clan members and are expected to play the intermediary role between the community and the administration. Respondents were selected using cluster sampling. Each *mji kumi* or a village elder’s area of jurisdiction formed a cluster for this study. A skip pattern was used to select households for the 423 quantitative questionnaires while participants for the focus group discussions (FGDs) comprised of all stakeholders (community leaders, political and religious leaders).

### Methodology

The study employed both qualitative and quantitative methods. Qualitative data facilitated interpretation of quantitative results in the context of the respondents’ daily lives and the role their knowledge, attitude and practices play in shaping their responses to industrial pollution. All respondents provided written informed consent before the questionnaires were administered. For the FGDs, group oral informed consent was obtained from participants; an oral statement was read to them and their agreement was indicated by a check mark on the consent form before commencing the discussion. We opted for oral consenting because we believed the topic of discussion involved no more than minimal risk to the FGD participants.

The qualitative interviews conducted after completion of the survey provided data that permitted corroboration and evidence on the impact of the factory closure. We took comprehensive notes of each FGD and they served as the textual basis for qualitative analysis.

### Data collection

The quantitative survey instrument was a questionnaire developed primarily by reviewing and adapting questionnaires from literature. For environmental perception, literature reviewed included previous risk perception, environmental health and environmental psychometric studies/ surveys within and outside Kenya which were then modified for relevance [3,17-30]. The survey questionnaire consisted of a list of items reported in literature as known or potential environmental health hazards from industrial facilities and environmental health perception indicators. Questions were developed based on variables representing aspects of perceived health risks identified from the above literature pertinent to the study area and population.

### Quantitative data management and analysis

The survey asked respondents to identify and describe the main risks from the paper mill in their neighborhood. Additionally respondents were asked to list and characterize the specific health and environmental risks they believed were associated with the paper mill.

Perception predictor variables were based on Vlek and Stallen’s personal decision-making model of risk acceptability and Riechard & Peterson perception questionnaire [26,31]. Found within that model are 32 “aspects of risk” categorized and believed to influence risk perception. Some elements like global warming, radon gas and asbestos were omitted based on the fact that majority of the respondents were likely to be unfamiliar with them. Also, Riechard & Peterson’s original questionnaire contained single 6-point Likert-type scale of environmental perception where as the scale here has been reduced to four or three-point (latter collapsed to two for logistic regression) and modified to scales of perceptions of exposure, benefits and control. The final list contained 20 common environmental variables.

The perceptions of control questions were adapted from the works of Riechard & Peterson, Schmidt & Gifford, Slovic and Westmoreland [26,27,32,33]. The same hazards were used for perception of exposure, severity and control. Benefit items were arrived at after reviewing the works of Gregory & Mendelsohn, Hallman and Hallman & Wandersman [23,24,34]. Thus literature on factors influencing the perception of (health) risks associated with exposure to environmental contaminants informed the selection of these variables. Respondents’ general risk perception was measured by asking them to rank their perception of risk for common environmental and health hazards in their neighborhood and other known environmental pollutants and health threats.

To “asses the residents’ awareness and beliefs concerning environmental risk especially their perception of air pollution health risk relative to other public health and environmental issues in their neighborhoods”, respondents’ responses to the 20 common environmental variables were compared across sub-locations. The 20 public health and environmental perception and control items were adopted from other environmental risk perception studies but modified for relevance and application to the study population [26,31-33]. Respondents were asked to rank their perception of risk (and control) of the 20 common risks on Likert-type scales; 1 (low risk), 2 (some risk) and 3 (high risk) that represented the amount of risk of harm/control they perceived for themselves, family and/or community from each hazard.

Raw data was cleaned by sorting out, grouping and coding of the completed questionnaires. The new data was cleaned by sorting out, grouping and coding of the completed questionnaires. Questionnaires were scrutinized for dishonesty and disregarded as necessary and answers to some interview questions helped in cross checking the consistency of responses. For example, in the early part of the interview respondents were asked the compositions of their household members (number of children and adults) and then in subsequent sections, they responded to another question on whether there were children with chronic illnesses in the household. If a respondent who had declared that there were no children in the household latter indicated that there were children with chronic illnesses in the household (as it happened in a few cases), then such data was deemed inconsistent and as a result the questionnaire was excluded from the analysis.

The quality of data was ensured through cross checking and computation before analysis. Respondent’s rating on each of the environmental hazard list was recorded.

All quantitative analyses were done using SPSS (Statistical Package for the Social Sciences) version 18. Bivariate analysis was performed to compare variables across sub-locations and to identify characteristics related to perceptions. Chi-square (χ^2^) test was used to test for the association, as all variables were categorical. Statistical levels of significance were set at 0.05. Multivariate analysis revealed factors associated with risk perception of the study population. Variables that were significantly associated with perceptions of risk and control were included in the final logistic regression analysis to confirm the most important factors influencing risk perception and environmental awareness in the communities while accounting for confounding.

Logistic regression was used to study the association of the original categorical independent variables with each of the five primary perception outcome (binary variables): - if respondent believes PPM has exposed him/her to hazardous chemicals; if the respondent felt he/she was coughing, breathless or wheezing due to something in the air; if the respondent believes the industry will affect children born in the future in the community; if the respondent believes the industry will expose him/her to hazards if it opens in the future; if the respondent worries about getting health problems in the future because of a polluted environment.

Whilst dichotomization is valid, it involves loss of information and there are fears that it may alter the findings. However, in an analysis of self-rated health and lifetime social class data, Manor et al., [35] compared dichotomized variable using logistic regression with alternative methods for ordered categorical variables and similar results and conclusions emerged from the five statistical approaches.

The covariates entered in all models included the following: the seven demographic factors, four household health indicators, six respiratory symptoms, two health risks characteristics, three benefit variables, four environmental awareness/knowledge variables and six information source variables (total 32 items) as predictors for air pollution as a health risk. All the above independent variables assumed to influence perception of health effects of air pollution were tested, following the stepwise procedure and the predictors that significantly improved the model were the only ones kept in the final step.

A total of 423 questionnaires were given out to respondents from the six sub-locations within the two districts (District A and District B) which fall within the five kilometer radius of the factory, the focus of this study. Three hundred and eighty two questionnaires were returned filled out and after thorough scrutiny 15 were discarded and the remaining 367 were entered in SPSS.

### Qualitative data management

Using Atlas.ti (version 5.2) software, the transcripts were coded, categorized and then grouped to generate larger environmental risk themes and subsequently correlated with the quantitative data analysis.

### Ethics statement

This study was conducted according to the principles expressed in the Declaration of Helsinki. The study was approved by the Institutional Review Board of Kenyatta National Hospital, Nairobi, Kenya, Ref: KNH/UON-ERC/A/249.

## Results and discussion

### Results

#### ***Descriptive statistics***

**Figure 2:**
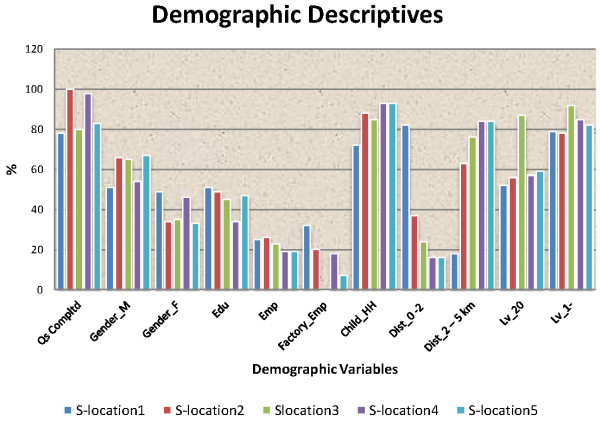
Demographic variables by sub-locations.

Table 1 and Figure 2 show demographic variables across the sub-locations, including the chi-square tests. They reveal differences in terms of household income, the presence of a child in the household, distance from the study factory and a family member having worked for the factory. This is expected as the sub-locations vary in distance from the town (where the factory is located) and population characteristics. The closure of the factory changed the population especially in the immediate vicinity of the town.

**Table 1:** Selected demographic characteristics of the study population

***District/Sub-location***		**Overall (n %)**	**District A**			**District B**		***p *** **value**
			**S-location 1**	**S-location 2**	**Slocation 3**	**S-location 4**	**S-location 5**	
Questionnaires	*Qs given*	423	143	50	50	110	70	
	*Qs Compltd*	367 *(87%)*	111 *(78%)*	50 *(100%)*	40 *(80%)*	108 *(98%)*	58 *(83%)*	
Gender	*M*	205 *(56%)*	54 *(49%)*	33 *(66%)*	26 *(65%)*	57 *(53%)*	35 *(60.5%)*	0.073
	*F*	154 *(42%)*	57 *(51%)*	17 *(34%)*	14 *(35%)*	49 *(45%)*	17 *(29%)*	
	*Missing*	8 (2%)				2 (2%)	6 (10.5%)	
Education (% Completed high school)		162 *(44%)*	56 *(51%)*	24 *(49%)*	18 *(45%)*	37 *(34%)*	27 *(47%)*	0.154
Employment (% Employed)		81 *(22%)*	28 *(25%)*	13 *(26%)*	9 *(23%)*	20 *(19%)*	11 *(19%)*	0.694
Household income	Up to 5,000	44 *(12%)*	5 *(5%)*	6 *(12%)*	1 *(3%)*	22 *(20%)*	10 *(17%)*	0.004*
	5,001-10,000	17 *(5%)*	4 *(4%)*	4 *(8%)*	0 *(0%)*	7 *(6%)*	2 *(3%)*	
	> 10,000	21 *(6%)*	8 (7%)	2 *(4%)*	1 *(3%)*	4 *(4%)*	6 *(10%)*	
	Missing	266 *(72%)*	92 *(83%)*	38 *(76%)*	38 *(95%)*	58 *(54%)*	40 *(69%)*	
	NA	19 *(5%)*	2 *(2%)*	0 *(0%)*	0 *(0%)*	17 *(16%)*	0 *(0%)*	
Family member worked for the factory		70 *(19%)*	36 *(32%)*	10 *(20%)*	2 *(5%)*	18 *(18%)*	4 *(7%)*	<0.001*
Child in household (%)		312 *(85%)*	80 *(72%)*	44 *(88%)*	34 *(85%)*	100 *(93%)*	54 *(93%)*	<0.001*
Distance from the factory	*0 – 2 km*	147 *(40%)*	93 *(84%)*	16 *(32%)*	10 *(25%)*	17 *(16%)*	11 *(16%)*	<0.001*
	*2 – 5 km*	220 *(60%)*	18 *(16%)*	34 *(68%)*	30 *(75%)*	91 *(84%)*	47 *(84%)*	
Lived there for >20 years		213 *(59%)*	57 *(52%)*	28 *(56%)*	34 *(87%)*	62 *(57%)*	32 *(59%)*	0.004*
Lived >10 years		286 *(83%)*	75 *(79%)*	39 *(78%)*	36 *(92%)*	92 *(85%)*	44 *(82%)*	0.315

Legend: Qs given: No of Questionnaires given out.

Qs Compltd: No. of Questionnaires completed.

*p-value ≤ 0.05.

No statistically significant difference was observed for gender, education and duration of stay of less than 20 years. Only 28% of respondents were able to estimate their household income.

#### ***Gender***

There were slightly more males than females in all the sub-locations except in Sub-location 1 where it was nearly balanced (51% males and 49% females).

#### ***Employment status***

Overall only 81 (22%) respondents reported being in employment at the time of the survey with the highest employment rate reported in Sub-location 3 (26%) and Sub-location 1 (25%). Sub-location 4 and Sub-location 5 had the lowest at 19%.

#### ***Occupation***

Occupations included small scale/informal businesses, teaching, clerical jobs and health professionals (doctors and nurses) with majority of respondents (21%; n = 75) being small scale farmers (peasant).

#### ***Employment with the factory***

**Figure 3:**
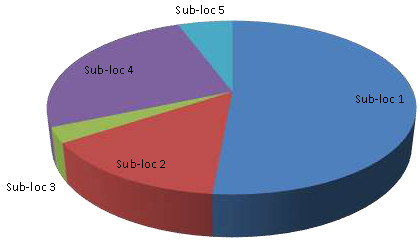
Employment with the study factory by sub-location.

Only 70 (19.1%) respondents had worked for the factory or had a member of their families who did. This was confirmed by the focus group discussions (FGDs). Surprisingly a very small proportion of the respondents had worked for the factory and even then those who had worked did so through subcontractors. Apparently very few employees were employed directly by the factory; instead the practice was to use subcontractors (out sourcing) as service providers to the factory through which a small number of residents were employed. The factory was situated in sub-location 1 and therefore this location had the highest proportion of respondents (and relatives) who were former factory employees (Figure 3).

#### Education

44% of the study respondents had completed high school and above compared to 22% of all Kenyans according to the 2008/09 Kenya Demographic and Health Survey -KDHS [36].

### Environmental awareness and risk perception

The summary of proportions of all respondents (combined) scores for medium to high levels of risk perceptions and control for the items are presented in Table 2. More than half of the respondents perceived all the elements as medium to high risk (with the exception of flooding).

**Table 2:** Perception and control of 20 environmental health risks and association (perception and control) of variables with air pollution perception

**Risk factors**	**Perception of risk and control**		**Association with air pollution perception ( *p *-value)**	
	**Reporting medium or high risk**	**Reporting medium or high control**	**Perception of risk**	**Perception of control**
Overpopulation	218 *(63%)*	212 *(62%)*	<0.001*	0.185
Destruction of Forests	254 *(75%)*	211 *64%)*	0.028*	0.451
Water Pollution	235 *(68%)*	214 *(64%)*	<0.001*	0.118
Air Pollution	275 *(82%*)	156 *(48%)*	**-**	<0.001*
Industrial Noise	246 *(73%)*	144 *(44%)*	<0.001*	0.526
Industrial Odor	271 *(81%)*	157 *(48%)*	<0.001*	0.453
Industrial Dust/Smoke	271 *(82%)*	168 *(53%)*	<0.001*	0.288
Motor Vehicle Accidents	233 *(70%*)	180 *(57%)*	0.040*	0.288
Motor Vehicle Pollution	197 *(61%*)	176 *(55%)*	<0.001*	0.561
Poor Housing	209 *(65%)*	200 *(63%)*	<0.001*	0.036*
Cooking with Firewood	191 *(57%)*	178 *(55%)*	0.113	0.515
Agricultural Chemicals	187 *(56%)*	188 *(58%)*	<0.001*	0.454
Waste/Plastic Bags	215 *(66%)*	200 *(63%)*	0.003*	0.230
Cigarette smoking	270 *(80%)*	235 *(72%)*	0.019*	0.560
Working Conditions	194 *(62%)*	167 *(55%*)	<0.001*	0.284
HIV/AIDS	269 *(80%)*	217 *(67%)*	0.002*	0.320
Drought	189 *(58%)*	169 *(55%)*	0.341	0.297
Famine	205 *(63%)*	183 *(59%)*	0.010*	*0.057*
Flooding	104 *(33%)*	171 *(57%)*	0.106	0.507
Soil Erosion	166 *(51%)*	214 *(68%)*	*0.055*	0.513

*p-value ≤ 0.05.

#### ***Risk perception***

Air pollution, industrial dust/smoke, cigarette smoking, HIV/AIDS and water pollution were perceived as the greatest risks. Flooding was perceived as the least risk and this is because the study area hardly experiences flooding.

#### ***Perception of control***

In all the 20 environmental and public health variables, the majority of the respondents reported low levels of control. Between 44% and 72% respondents felt they had medium/high level of control over the range of listed environmental factors with majority being in the 50s/60s. Respondents had the highest perception of control over cigarette smoking followed by perception of control over soil erosion (68%). On the other hand respondents reported the least level of control over industrial noise (44%), odor and air pollution (48%).

#### ***Association between risk variables***

The second set of analysis involved bivaraite analysis to test the associations between air pollution perception (dependent) with the other environmental and public health variables. Table 3 shows these analysis results.

i). *Association of Air Pollution Perception with Perceptions of other 19 Environmental Risk variables*

Significant associations were found between perception of air pollution as a risk and all the listed environmental threats except for perceptions of drought, flooding and soil erosion as risks. Thus fifteen of the 19 perception variables were found to be significantly associated with air pollution with nine of them being highly significant; p < 0.001

ii). *Air Pollution Perception* versus *Perception of Control of other variables*

Only the association between perception of air pollution and perception of control over air pollution was found to be highly significant (*p* < 0.001). The remaining perception variables were insignificant except for perception of control of famine with borderline significance, *p* = 0.0507.

Overall respondents perceived air pollution as a risk. For example out of the respondents who thought overpopulation was a high risk, the majority (74%, *n* = 78) perceived air pollution as a risk and even those who perceived overpopulation as a low risk, majority still perceived air pollution as a high risk (48%, *n* = 60). The same general trend (i.e. majority perceiving air pollution as a high risk) was observed across all the twenty perception variables with a couple of exceptions - drought and flooding - both of which are typically not a problem in the study area.

**Table 3:** Final logistic regression models

		**Believes PPM has exposed him/her to hazardous chemicals**		**Felt he/she was coughing, breathless or wheezing due to something in the air**		**Believes the industry will affect children born in the future in the community**		**Believes the industry will expose him/her to hazards if it opens in the future**		**Worries about getting health problems in the future because of a polluted environment**	
		**OR**	**95% CI**	**OR**	**95% CI**	**OR**	**95% CI**	**OR**	**95% CI**	**OR**	**95% CI**
Demographic	Distance from PPM	2.747*	1.199-6.295	1.039^‡^	0.560-1927	1.132^‡^	0.502-2.554				
	Presence of a child in the HH	34.769*	1.390-869.96								
	Respondent completed HS	3.105*	1.394-6.918							2.553*	1.086-6.004
	Employment Status					0.434^‡^	0.188-1.004	0.383*	0.167-0.879	0.365*	0.150-0.886
HH health Indicators	Perception of health	0.527^‡^	0.239-1.162	0.479*	0.261-0.878	0.299*	0.135-0.660	0.237**	0.108-0.517	0.332*	0.149-0.738
	Somebody in the HH has one of the conditions (heart, respiratory or skin disease)	1.788^‡^	0.755-4.237	1.980*	1.031-3.802	2.192^‡^	0.963-4.990			2.594*	1.096-6.143
Respiratory symptoms	Coughed phlegm daily for ≥ 2 months, 2 yrs in a row			2.907*	1.127-7.498						
	Had an attack of whistling or noisy sound in the chest when breathing			2.881*	1.402-5.920						
Role of Industrial plant	Family depended on industry					2.287*	1.001-5.228	2.792*	1.234-6.319		
	Community benefited from industry									2.289*	0.997-5.257
Environmental awareness/knowledge	Ever actively looked for environmental health information			1.883*	1.002-3.537						
	Willing to participate in environmental campaigns			2.297^‡^	0.713-7.396	2.985*	1.031-8.645			3.127*	1.100-8.888
Main Information source	Friends/relatives	2.553*	1.198-5.438			0.411*	0.180-0.937	0.451*	0.207-0.981		
	Church/Community leaders					3.148*	1.303-7.602	2.368*	1.090-5.147	2.447*	1.089-5.499
Omnibus Tests of Model Coefficients	Model (Sig.)	<0.001		<0.001		<0.001		<0.001		<0.001	
Correctly predicted observations on average		85.6%		79.7%		84.6%		86%		84.4%	

^‡^p > 0.05; *p < 0.05; **p < 0.01; HH - Household: HS - High school.

### Multivariate analysis

A summary of the logistic regression models predicting respondents’ perceptions of the health effects of PPM pollution and the main predictor factors of three of these perceptions are summarized in Table 3. The table shows the confidence intervals, the odds ratios and *p-values* of each remaining predictor in the respective models as well as the percentages of correctly predicted observations for each model.

Based on the variables that emerged significant in this model, factors that influence the residents’ belief that *PPM had exposed them to hazardous chemicals* are; distance from PPM, presence of a child in the HH, perception of health (though not significant) education level, presence of a smoker in the HH, perceived benefits from PPM and friends/relatives being the main source of information.

The odds ratio showed that compared to those who live far, those who live near the factory were almost 3 times more likely to believe that PPM had exposed them to hazardous chemicals. Similarly, families with children are 35 times were more likely to believe they had been exposed; those who had completed high school 3 times more likely to believe they had been exposed; those who rely on friends/family as the information source are 2.5 times more likely to believe PPM had exposed them to hazardous chemicals.

The ‘*Respondent believes PPM has exposed him/her to hazardous chemicals’* model was able to correctly predict 85.6% of observations, from the Omnibus Tests of Model Coefficients table the model significance level was <0.001 showing that hat the final model predicted the dependent variable. The results are consistent with the descriptive and bivariate results showing that respondents generally perceive the paper mill as a major source of pollution.

For the perception: *Respondent felt he/she was coughing, breathless or wheezing due to something in the air,* the overall model was able to correctly predicted 79.7% of observations. The model significance level was <0.001, showing that the independent variables are associated with the dependent variable. Respondents had somebody in the household with one of the conditions (respiratory, heart or skin conditions), those who coughed phlegm for at least two months for two years, those who had experienced an attack of whistling or noisy breathing and those who had looked for environmental health information were more likely to have sensed respiratory irritants in the air (i.e. participant felt he/she was coughing, breathless or wheezing due to something in the air). On the other hand, respondents who view their health as good/excellent (compared to those who perceive their health as poor/fair) are less likely to say they had sensed respiratory irritants in the air.

Respondents who perceive their health is excellent/good were less likely to say the industry will affect children born in the future in the community. On the other hand, respondents who viewed their health as poor/fair; were willing to participate in environmental campaigns, those whose families benefited on PPM, and those who rely on the church as the main source of information were more likely to *believe the industry will affect children born in the future*. From the Omnibus Tests of Model Coefficients table the final model for ‘*Respondent believes the industry will affect children born in the future in the community’* had a p-value < 0.001 and was able to correctly predict 84.6% of observations.

Participants who were employed and those who viewed their health as good/excellent were less likely to believe the *industry will expose them to hazards if it opens in the future*. The model was able to correctly predict 86% of observations.

Likewise, employed participants and participants who view their health as good/excellent were less likely to *worry about getting health problems in the future due to a polluted environment*. On the other hand participants who - had completed high school, knew somebody who is affected, were willing to participate in environmental campaigns and those who believed the community benefited from PPM and relied on community leaders for their information were more likely to *worry about getting health problems in the future due to a polluted environment*. The model is highly significant, showing that the independent variables predict the dependent variable well and was able to correctly predict 84.4% of observations.

### Discussion

The qualitative interviews conducted after completion of the quantitative survey provided data that permitted corroboration of quantitative data and evidence about the impact of the factory closure.

A large proportion of respondents were unemployed hence the closure of the factory may have negatively affected the economy of the study area. It is worth noting that majority of respondents (81%, n = 297) had not worked for the factory nor their family members and only 19% (n = 70) of respondents had a family member whom at one time worked for the factory. This sentiment was confirmed during the FGDs where respondents claimed the factory had mostly benefited foreigners; that the locals were rarely employed under the pretext that the locals were uneducated. While most respondents said the factory had not employed locals, the presence of the factory supported other economic activities in the region as confirmed by the FGDs, e.g. ready markets for their agricultural products and income from rental houses among others.

There were mixed reactions to the environmental effects of the factory closure. While some participants said there was no difference, most of them felt the environment (and agricultural production) had improved;

*“… since the factory closed, I planted cassava and am seeing prospects of having a good cassava harvest, compared to when the factory was operating… even vegetables*” a village elder from Sub-location 5 said.

#### ***Awareness and perception***

In this study, efforts were made to examine perceptions of risks around actual hazards that respondents had experienced or at least could relate to. Other researchers have also examined perceptions of risk around specific hazardous activities/technologies in affected communities [24,37]. Respondents in this study rated the risk of harm from a list of common environmental and public health hazards by indicating the extent to which they believed these hazards posed serious health threats to themselves, their family members’ and the community. The results revealed high risk perception for the hazards that the respondents are familiar with and commonly exposed to. With the exception of flooding, overall majority (over 50%) off the respondents perceived all the listed environmental/health factors as medium or high risk. Air pollution, industrial dust/smoke (82%), industrial odor (81%) and HIV/AIDS & cigarette smoking (80%) were perceived to have the highest risk factors in the list (Table 2).

Risks considered to be involuntary like industrial hazards are generally dreaded because they are viewed as being more dangerous than those that are more familiar or voluntary [38-40]. In many previous environmental studies, respondents were asked to rank hazards, some of which had no relevance to them and therefore posed no threat as far as they were concerned. This study only included threats applicable and known to the study community.

The hazards like HIV/AIDS, air and water pollution and motor vehicle accidents that were ranked highly in this study, have already been identified in other studies to be perceived as high risk by Kenyans and are often mentioned as major challenges facing Kenya as a country [41,42].

Generally the risk perception of all respondents reflected a universal concern for pollution. This finding contradicts the widely predicted view that low income individuals/communities have more pressing problems and do not have the ‘luxury’ or the time and knowledge to worry about pollution of their environment [43,44]. As revealed by the FGDs, residents of the study area did not just accept the status quo. On the contrary, there had been many attempts to address the pollution problem but unfortunately lack of proper representation by local leaders, personal interests of some leaders and political interference were identified as some of the barriers to solving the pollution problem. As a result of the perennial non-response to their many complaints, the residents have developed some apathy and belief that nobody cares about their problems (pragmatic acceptance). This came out repeatedly in the FGDs. Some environmental perceptions studies have shown that low income populations are well aware of their increased vulnerability to environmental hazards but feel disempowered to act appropriately and with time, they embrace their disadvantaged circumstances [45,46].

Other perception studies have revealed different views on environmental issues by rural and urban residents with rural populations having a tendency to focus on community economic benefits of activities/technology over environmental management and protection of natural resources [47]. This study revealed a scenario where the economic benefit of the factory to the residents was mainly derived from business prospects and not from direct employment opportunities as in the studies reviewed. This may explain why they were not shy to express their fears about the negative effects of the factory on probing.

Generally cigarette smoking is rare in rural areas in Kenya hence its high perception of as a health risk in this study. According to WHO (2002) annual cigarette consumption per person in Kenya is less than 500 [48]. Accordingly, respondents reported the highest level of control (72%) over this environmental risk. Similarly the high perception of HIV/AIDS as a health risk is in line with the disease epidemic in Kenya; currently 1.4 – 1.6 million Kenyans are estimated to be living with HIV/AIDS [35,49].

The perception of flooding as a low risk can be explained by the fact that flooding is a very rare phenomenon in the study area. Consequently respondents reported a relatively high level of control over this risk (67%) but reported least levels of control over air pollution; industrial noise and industrial odor, and perceived them as high risks.

The findings above demonstrate relatively high levels of awareness and concern for environmental pollution. Compared to other environmental risks, air pollution is definitely a major concern for the study town residents where over 80% of the residents perceived it as a high risk.

Only 34% residents perceived flooding as a risk and the perception association between flooding and of air pollution was insignificant. This can be explained by the fact that flooding is not a common occurrence in this region and therefore not one of the problems they worry about. About half of the respondents (51%) perceived soil erosion as a risk but 68% felt they had control over the hazard. Perception association of this hazard with air pollution was insignificant.

#### ***Association between perception of air pollution and perception of control***

Literature on risk perception reveals that the ability of individuals to influence circumstances that affect them is closely tied to perceptions of risk and if they feel they have control over a risk then they perceive it as a low risk and vice versa [50,51]. This phenomenon is contradicted by the bivariate analysis of perception of air pollution and perception of control over the same. Of the respondents who perceived air pollution as a high risk, majority (about 55%) reported having some control over it and 51% of respondents who reported low risk felt they had low level of control.

It is also worth noting that out of those who felt they had a high level of control over air pollution (48%, n = 156), majority (70.5%, n = 109), perceived air pollution as a high risk. So, generally air pollution was perceived as a high risk irrespective of the respondents’ perception of control. The association between these two measures of perception was highly significant. The association of air pollution with perceived ability to deal with the problem has been demonstrated in other studies [52,53]. The public has a tendency to feel their actions have little or no impact as far as reducing air pollution is concerned and this perceived low level of control over environmental problems may discourage affected populations from becoming part of the solution.

From the FGDs there was a general belief that other stakeholders (political leaders, management of industries, government officials) were unable to act responsibly to alleviate the pollution problems and some respondents further indicated that it was not their responsibility because they did not have the capacity to effectively act to protect their environment. Most of the respondents had lost faith in the government regulatory agencies, and were of the opinion that the government officers receive bribes to defend the interests of the factory management.

Health status and distance from a polluting facility have been shown to be a strong predictor of risk perception. A couple of studies done in India - a developing country - revealed that persons neighboring industrial settings are twice more likely to experience respiratory symptoms/illness compared to those living far away [6,31]. Obviously people who live close by bear the burden of the pollution (odor, smoke, dust, etc.) and naturally believe it is affecting their health. Participants were able to clearly narrate the history of the paper mill during the FGDs. Those close to the factory reported that the arrival of the factory marked the beginning of diminishing agricultural outputs. However in the logistic regression models, it was a modest a predictor of environmental risk perception as one of the models predicted that people who live closer to the factory are over twice more likely to believe PPM had exposed them to hazardous chemicals. Also from the logistic regression models, presence of a child in the household was a predictor for one perception variable; belief of PPM was a source of exposure to hazardous chemicals. Existing literature has confirmed that the connection between environmental exposure and possible health effects is mostly attributed to concerns over children’s health [20,54,55].

The logistic regression models further confirmed the MLR finding that family health status was the best predictor of environmental risk perception. Family health status variables were predictors in four out of the six models - belief that PPM exposed residents to hazardous chemicals, experience of coughing, breathless or wheezing due to something in the air, belief that the industry will affect children born in the future in the community, the belief that the industry will expose him/her to hazards if it opens in the future and worrying about getting health problems in the future because of a polluted environment.

Out of the health status variable, only the perception variable ‘*respondents’ perceptions of his/her health’* turned out to be a strong predictor for environmental risk perception in this study. The other family health status variables like presence of an unwell child in the household did not come out clearly as predictors for environmental risk perception. The respondent’s view of health was a predictor for four out of the seven dependent risk perception variables (the respondent believing the industry will affect children born in the future in the community; the respondent sensing respiratory irritants in the air; the respondent believing the industry will expose them to hazards if it opens in the future and respondent worrying about getting health problems in the future because of a polluted environment). People who perceived their health were found to be less likely to have a high perception of environmental hazards. This can only be attributed to the fact that because they feel healthy, they do not seem to see the danger around them. And have a tendency to be complacent.

## Conclusion

Study respondents clearly demonstrated their awareness and concern over negative effects of air pollution as well as other environmental and agricultural activities on their health. Although public perceptions are influenced by many factors, the concerns are consistent and call for involvement of the affected individuals – their social status notwithstanding – in environmental management and policy formulation. This study presented a quantitative approach to environmental risk perception. It examined pertinent environmental hazards to generate fundamental information on environmental beliefs within the study community through both closed and open ended questions. With increased awareness, individual involvement and support, participation by rural communities could be central to achieving environmentally friendly and sustainable industrial development in emerging economies.

The findings will enable the stakeholders, (the researcher, the public, the industry and the policy makers) to focus on what is important in mediating the pollution risks in the community as environmental policies are developed.

### Limitations of the study

This was one of the very few studies in Kenya that attempted to assess community perception of pollution; however, it had following limitations:

• The study area was limited to 5 km radius of the paper mill

• The factory closed down due to financial problems implying views of the long-term residents and regular workers of the factory were not captured.

• Lack of health facility data to confirm or contradict respondents’ claims.

• The fact that most respondents felt the factory did not benefit them as much as ‘foreigners’ may have biased the study.

## Abbreviations

AIDS: Acquired Immunodeficiency Syndrome; FGD: Focus group discussions; HIV: Human immunodeficiency virus; KDHS: Kenya Demographic and Health Survey; MoH: Ministry of health; NASCOP: National AIDS & STI Control Program; NEMA: National Environment Management Authority; UNEP: United Nations Environment program; WHO: World Health Organization.

## Competing interests

The authors declare that they have no competing interests.

## Authors’ contributions

EO: Conceived the study and designed; carried out the field data collection and data entry; performed statistical analysis; drafted the manuscript. LU: Guided the conception and design, supervision of the research, helped with manuscript draft and revision. ZB: Guided statistical analysis, helped with manuscript draft. MG: Guided field data collection, helped with manuscript draft. All authors have given final approval of this version to be published.

## Authors’ information

EO (DrPH): Head of Research, Impact Research and Development Organization.

LU (PhD): Chair, Community Health and Development Department, Drexel School of Public Health.

ZB (PhD): Prof. Epidemiology and Biostatistics department, Drexel School of Public Health.

MG (PhD): Senior Lecturer, Institute of Nuclear Science and Technology, University of Nairobi.

## Pre-publication history

The pre-publication history for this paper can be accessed here:

http://www.biomedcentral.com/1471-2458/14/377/prepub
